# First meta-analysis study of cholinesterase inhibition in experimental animals by organophosphate or carbamate insecticides under the influence of diphenhydramine

**DOI:** 10.14202/vetworld.2023.118-125

**Published:** 2023-01-16

**Authors:** Fouad K. Mohammad, Hishyar M. S. Garmavy, Ammar A. Mohammed, Hussein M. Rashid

**Affiliations:** 1 Department of Physiology, Biochemistry, and Pharmacology, College of Veterinary Medicine, University of Mosul, Mosul, Iraq; 2 Department of Pharmacology, College of Pharmacy, University of Duhok, Duhok, KRG, Iraq

**Keywords:** acetylcholinesterase, antihistamine, carbaryl, dichlorvos, meta-analysis, methomyl

## Abstract

**Background and Aim:**

Diphenhydramine is an H1-antihistamine that counteracts the toxic effects of organophosphate and carbamate insecticides that inhibit cholinesterase (ChE) activity. This meta-analysis aimed to investigate the effects of diphenhydramine on ChE inhibition induced by these insecticides in the plasma, erythrocytes, or whole brain of experimental animals.

**Materials and Methods:**

A data search was performed on erythrocyte, plasma, and brain ChE inhibition caused by organophosphate and carbamate insecticides in experimental animals (mice, rats, and chicks) treated with the antihistamine diphenhydramine in accordance with preferred reporting items for systematic reviews and meta-analysis, which was done by the two-group random-effects model meta-analysis. The meta-analysis included 18 records extracted from six studies that, appeared from 1996 to 2022.

**Results:**

Using the random-effects model, a two-group meta-analysis revealed that the combined effect size (ChE inhibition) was significantly more favorable in the control group than in the diphenhydramine intervention, as shown by a forest plot. The combined effect size (standardized mean difference) was 0.67, with a standard error of 0.3, a lower limit of 0.04, and an upper limit of 1.29 (p = 0.025). The heterogeneity was moderate, as I^2^ of the combined effect size was 74%, with a significant Cochrane Q-test result (Q = 65, p < 0.0001). Subgroup analysis indicated that, with brain ChE inhibition, the heterogeneity (I^2^) became 5%, which was lower than ChE inhibition in plasma (84%) and erythrocytes (78%). No publication bias was identified using the funnel plot and Egger’s test.

**Conclusion:**

This meta-analysis suggests that, in addition to its documented antidotal action against ChE-inhibiting insecticides, diphenhydramine can also reduce the extent of ChE inhibition, especially in the brain, which is the main site of toxicity of these insecticides. There is a need for additional studies to assess such enzyme inhibition in different parts of the brain.

## Introduction

In veterinary clinical practice, public health, and agriculture, there is a widespread use of formulations of organophosphate (OP) or carbamate (CA) insecticides to control pests [1–3]. Such insecticides can result in poisoning by inhibiting the key enzyme cholinesterase (ChE) at the nerve terminals, leading to cholinergic overstimulation with subsequent effects on muscarinic, nicotinic, and central nervous systems and the production of a cascade of events potentially resulting in respiratory failure and death [4–6]. However, the inhibition of ChE activity by OP is irreversible, whereas that by CA is reversible [4, 6]. Based on the properties of these insecticides in inhibiting ChE, ChE activities in blood, including those in serum or plasma cholinesterase (PChE) and erythrocytes cholinesterase (EChE), are usually measured as biomonitoring of the exposure to pesticides and to assess the status of poisoning [7, 8].

Diphenhydramine is an H1-antihistamine that counteracts the toxic effects of OP and CA insecticides [9, 10]. Several studies in mice [11–13], rats [14–19], and chicks [20–22] have clearly demonstrated that diphenhydramine, similar to the standard antidote atropine, reduced the toxicity of these insecticides. The antidotal or preventive actions of diphenhydramine against these insecticides involved increasing the median lethal insecticide dose, decreasing the occurrence of cholinergic toxidrome with reductions in toxicity scores, delaying the latency to poisoning onset and death, and increasing the survival rate [11–13, 16, 21, 22]. It has also been reported that diphenhydramine reduced OP-induced injuries in the pancreas [18] and heart [19] of laboratory rats. Clinically, diphenhydramine has been used in cases of poisoning by OP or CA insecticide in humans [23, 24], dogs [25–27], cats [28], and chickens [29]. Review articles have also been published on the beneficial effects of this antihistamine against pesticide poisoning in animals [9, 10].

Regarding the mechanism behind the antidotal action of diphenhydramine against OP or CA insecticide, its antimuscarinic and possibly antinicotinic properties have been highlighted [9, 10, 22, 23]. However, there is no clear understanding of the interaction of diphenhydramine with antiChE insecticides at the level of the ChE. By examining OP-or CA-induced inhibition of ChE in the blood (PChE and EChE) or whole-brain cholinesterase (BChE) following *in vivo* animal experiments, it was reported that diphenhydramine did not affect the extent of ChE inhibition in rats [14, 15] and mice [13], whereas the drug reduced the ChE inhibitory effects of OP and CA in chicks [20–22]. It was also reported that, *in vitro*, diphenhydramine partially reduced the inhibitory actions of the CA insecticide carbaryl on PChE and EChE in humans [30] and on PChE and BChE in chicks [22]. However, one study performed in mice indicated that PChE inhibition was increased by the combination of diphenhydramine and OP dichlorvos [31]. Meanwhile, diphenhydramine alone was reported to exhibit inhibitory effects on PChE and BChE *in vivo* in chicks [32]. These findings raised the possibility that diphenhydramine could weakly inhibit OP-induced ChE inhibition [21, 22, 30], as found with other weak ChE inhibitors [33, 34].

Against this background, and because of the various, although conflicting, studies on ChE activities under the influence of diphenhydramine with OP or CA insecticides, this meta-analysis was established to investigate for the 1^st^ time the effects of antihistamine on ChE inhibition induced by OP (dichlorvos) or CA (carbaryl, methomyl) insecticides, as found in the literature.

## Materials and Methods

### Ethical approval

This is a meta-analysis, which was approved by the Reviewing Board, College of Pharmacy, University of Duhok, Iraq, and no live animals were used.

### Study period and location

The study was conducted from January 2, 2022, to July 31, 2022, at the College of Pharmacy, University of Duhok, Iraq.

### Search strategy

The data search was based on research articles and academic theses published from 1980 to July 31, 2022, on the combined effects of diphenhydramine with OP or CA insecticides in experimental animals (mice, rats, chicks), using PubMed, Google Scholars, Scopus (Science Direct), Directory of Open Access Journals, Semantic Scholar, Web of Science, My Research Assistant, and Iraqi Academic Scientific Journals databases. A manual search was performed on articles and academic theses from Iraqi universities because most of these resources do not appear in the above-mentioned databases. No language restriction was applied to the search strategy.

### Selection criteria

**Figure-1:**
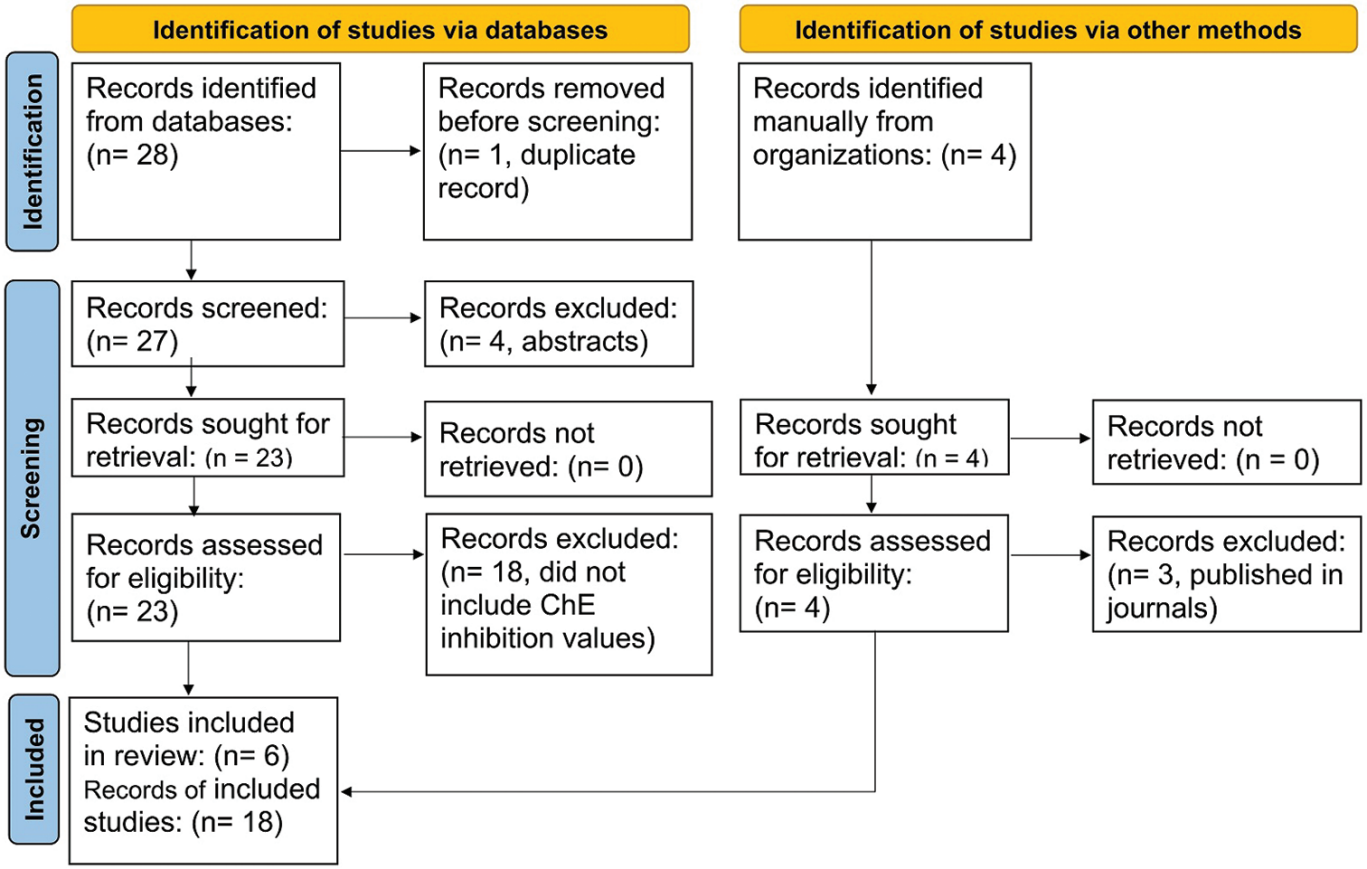
Preferred reporting items for systematic reviews and meta-analysis flow diagram for a systematic review of studies, including searches of databases and other sources recording blood or brain cholinesterase inhibition in experimental animals intoxicated with organophosphate or carbamate insecticides and treated with diphenhydramine.

As shown in Figure-1, 18 records were extracted from six studies on the inhibitory effects of OP or CA insecticides on PChE, EChE or BChE activities in experimental animals subjected to various treatment regimens with diphenhydramine. The records were selected in accordance with the Preferred Reporting Items for Systematic Reviews and Meta-Analysis (PRISMA) [35]. The selected papers included experimentally obtained values of blood or tissue ChE activities (mean, standard deviation, and standard error) under the *in vivo* influence of OP or CA insecticides with concurrent diphenhydramine (experimental intervention) or saline (control) treatment. The diphenhydramine treatments were administered either before or after the OP or CA insecticide dosing.

### Inclusion and exclusion criteria

Studies included in the meta-analysis were those reporting effects of diphenhydramine on ChE activities in blood or tissue in experimental animals treated with OP or CA insecticide. Records were excluded if they were from studies using antihistamines other than diphenhydramine or did not report ChE activity in experimental animals. *In vitro* studies were also excluded from the study.

### Data extraction

Data on ChE activities, as described in the selection criteria, were extracted from the texts, tables, or figures of the identified manuscripts. They reported ChE inhibition in experimental animals subjected to OP or CA insecticide treatments with or without diphenhydramine therapy. Some papers reported more than one subset of ChE inhibition data; such data were also included in the meta-analysis. We calculated the standard deviations from the corresponding standard errors depending on the data presented in the studies. When the numbers of experimental animals were presented as a range of minimum-maximum per group, the minimum number was chosen, to avoid increasing the favorable outcome toward the treatment effect. Any disagreement about data extraction was resolved by the authors of this paper through discussion.

### Statistical analysis

A two-group meta-analysis was conducted in accordance with PRISMA guidelines using the random-effects model because of the expected sources of heterogeneity among different studies (Table-1) [14, 15, 20–22, 31]. The meta-analysis was performed using the software Meta-Essentials Version 1.5 (https://www.erim.eur.nl/research-support/meta-essentials/download/), which included a forest plot to calculate the effect size, weighted means with their 95% confidence intervals, a funnel plot, and subgroup analysis [36]. Along with visualizing the meta-analysis as a forest plot, a drapery plot was produced using Meta-Mar software (https://www.meta-mar.com/) as further supporting evidence, with proposed confidence intervals at p < 0.05 [37].

**Table-1:** Cholinesterase activities (ΔpH/incubation time, 30 min) of experimental animals under the influence of organophosphate or carbamate insecticides with or without diphenhydramine treatment.

Reference	Year	Species	Code	ChE	OP/CA+Diphen			OP/CA-no Diphen		
	
					n	Mean	SD	n	Mean	SD
Faris and Mohammad [31]	1996	Mouse	A	PChE	4	0.154	0.062	4	0.308	0.158
			B	EChE	4	0.123	0.036	4	0.123	0.062
			C	Brain	4	0.237	0.044	4	0.22	0.052
Baggou and Mohammad [14]	1999	Rat	D	PChE	4	0.17	0.02	4	0.2	0.0276
			E	PChE	4	0.18	0.042	4	0.18	0.02
			F	EChE	4	0.25	0.024	4	0.182	0.024
			G	EChE	4	0.22	0.048	4	0.17	0.024
Mohammad *et al*. [15]	2002	Rat	H	PChE	5	0.08	0.0224	5	0.11	0.0447
			I	EChE	5	0.09	0.0224	5	0.12	0.0224
			J	Brain	5	0.13	0.0671	5	0.12	0.0447
Al-Shammary [20]	2008	Chick	K	PChE	8	0.11	0.017	8	0.08	0.0141
			L	Brain	8	0.16	0.2461	8	0.06	0.0028
Mohammad *et al*. [21]	2012	Chick	M	PChE	8	0.15	0.0764	8	0.008	0.0113
			N	PChE	8	0.16	0.0594	8	0.046	0.0368
			O	Brain	8	0.12	0.0283	8	0.11	0.0226
			P	Brain	8	0.2	0.0877	8	0.08	0.0311
Mohammed and Mohammad [22]	2022	Chick	Q	PChE	6	0.242	0.0122	6	0.205	0.0147
			R	Brain	6	0.1	0.0196	6	0.08	0.022

ChE=Cholinesterase, PChE=Plasma cholinesterase, EChE=Erythrocyte cholinesterase, BChE=Brain cholinesterase, OP=organophosphate, CA=Carbamate, diphen=Diphenhydramine, ChE activities were measured by a modified electrometric method

### Heterogeneity analysis

The Cochrane Q-test was used to statistically examine any heterogeneity [38–40] at a p-value threshold of < 0.10. In addition, I^2^ was calculated using the above-mentioned software (Meta-Essential), with values ranging from 0% (no heterogeneity) to 100% (high heterogeneity) [38–40].

### Publication bias

A funnel plot (size effect against standard error) was used to visually assess any publication bias, while Egger’s statistical test was applied to objectively confirm the existence of such bias [40, 41].

## Results

Figure-1 shows the PRISMA flow chart for selecting study records on blood or tissue (brain) ChE inhibition caused by OP or CA insecticides in experimental animals treated with diphenhydramine. Initially, the search strategy retrieved 32 studies involving diphenhydramine use, either therapeutically in humans or animals or experimentally in animals suffering from OP or CA poisoning. The final list of selected records (n = 18) for the meta-analysis included those from five articles and one thesis (Figure-1 and Table-1). They specifically involved inhibition of ChE activities in the blood or whole brain of experimental animals (mice, rats, and young chicks) under the influence of OP or CA insecticide with or without concomitant diphenhydramine therapy. These records were published between 1996 and 2022 (Table-1). The insecticides used in the selected studies were the OP dichlorvos in mice [31], rats [15], and chicks [20, 21], or the CA methomyl in rats [14] or carbaryl in chicks [22]. The cumulative number of experimental animals with OP or CA insecticide intoxication in the selected studies was 103 for the diphenhydramine treatment and also 103 for the control one (Table-1). The enzyme sources were PChE, EChE, and BChE, comprising 94, 34, and 78 samples, respectively (Table-1).

### Meta-analysis

**Figure-2:**
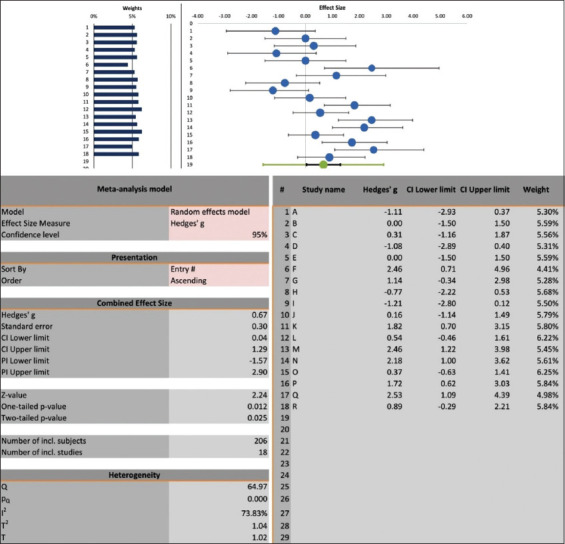
Forest plot of the records of comparison of cholinesterase inhibition in the plasma, erythrocytes or brain of experimental animals (mice, rats, and chicks) intoxicated with organophosphate or carbamate insecticides treated with diphenhydramine (left side of zero effect size) or with saline as untreated controls (right side of zero effect size).

Using the random-effects model, a two-group meta-analysis of these records revealed that the combined effect size (ChE inhibition) was significantly more favorable in the control group, as shown by the forest plot, indicating the value of diphenhydramine for treating OP or CA poisoning (Figure-2). This value was conferred via lower ChE inhibition than in the control group. The related statistics were as follows: combined effect size (standardized mean difference) 0.67, standard error 0.3, lower limit 0.04, upper limit 1.29, and Z value 2.24 with a two-tailed p-value of 0.025. The percentages of weights of individual records obtained from the forest plot were close to each other and ranged from 4.41% to 6.25% (Figure-2).

### Heterogeneity analysis

We used Cochrane Q-test and the I^2^ index to examine the heterogeneity among the studies of this random-effects model of meta-analysis (Figure-2). The heterogeneity I^2^ index (with T^2^ and T values of 1.04 and 1.02, respectively) of the combined effect size was 74%, indicating a moderate degree of heterogeneity, which was also confirmed to be statistically significant using the Cochrane Q-test (Q = 65, p < 0.0001).

### Drapery plot

**Figure-3:**
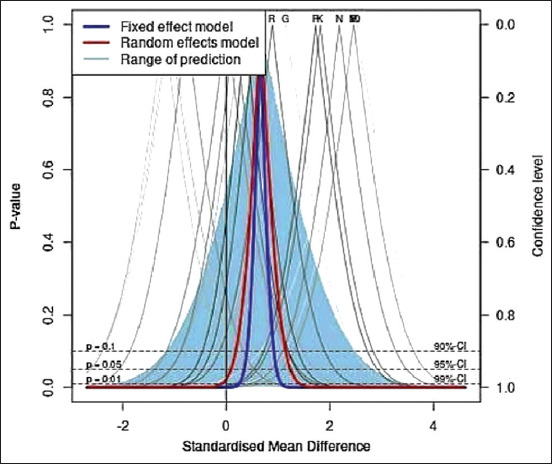
Drapery plot of the records of comparison of cholinesterase inhibition in the plasma, erythrocytes or brain of experimental animals (mice, rats, and chicks) intoxicated with organophosphate or carbamate insecticides treated with diphenhydramine or with saline as untreated controls. The plot shows p-value function (confidence curve) for individual records as well as meta-analysis estimates, and the shaded area depicts a prediction region for a single future study.

In addition to the forest plot, a drapery plot with proposed confidence intervals was applied to these data (using Meta-Mar software). The drapery plot (Figure-3) provided support for this meta-analysis based on the p-value functions of each record (confidence curve, Y axis = p values; X axis = effect size). Furthermore, Figure-3 shows meta-analysis estimates, and the shaded area depicts a prediction region for a single future study, suggesting the possibility of a shift of the point of effect size toward the diphenhydramine intervention.

### Subgroup analysis

**Figure-4:**
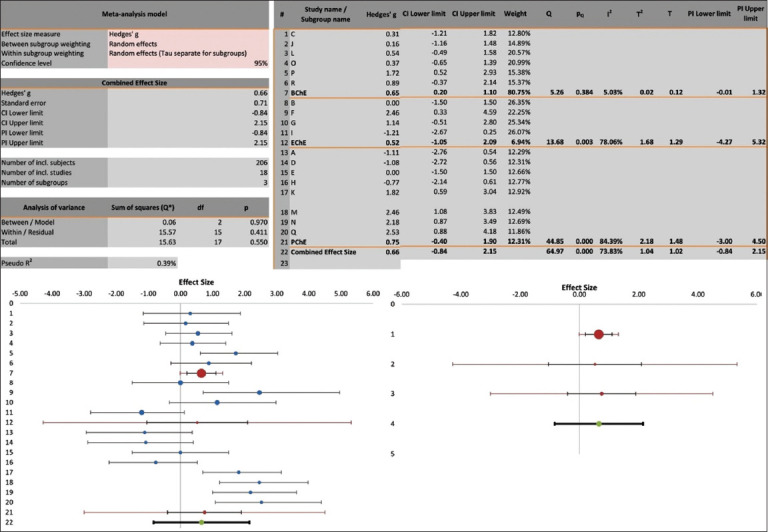
Subgroup analysis and related forest plots to identify the cause of heterogeneity of the records of comparison of cholinesterase inhibition in the plasma cholinesterase, erythrocytes cholinesterase, or brain cholinesterase of experimental animals (mice, rats, and chicks) intoxicated with organophosphate or carbamate insecticides and treated with diphenhydramine or with saline as untreated controls.

To identify the cause of heterogeneity, subgroup analysis was conducted. The related forest plot with the associated statistics is depicted in Figure-4. With BChE inhibition, the heterogeneity (I^2^) became 5% (Q = 5, p < 0.384), compared with equivalent PChE and EChE values of 84% (Q = 45, p < 0.0001) and 78% (Q = 14, p < 0.003), respectively. Correspondingly, the weight of BChE in the subgroup analysis was 81%, compared with 12% for PChE and 7% for EChE.

### Publication bias (funnel plot)

**Figure-5:**
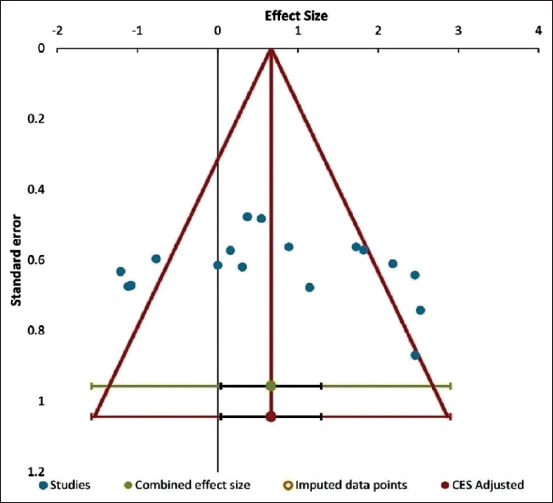
Funnel plot to identify any publication bias.

To examine whether there was publication bias, the funnel plot was visually examined. This indicated no publication bias, given the symmetrical distribution of the effect size points in a limited area of the plot (Figure-5). This conclusion was further objectively and statistically confirmed by Egger’s test, the result of which was not significant (t = 0.59, p = 0.56).

## Discussion

Studies in humans [23, 24] and animals [9, 25] have demonstrated that diphenhydramine can be used as an alternative, but not a substitute, antidote against poisoning induced by ChE-inhibiting OP or CA insecticides. The antidotal effects of diphenhydramine against poisoning by OP or CA insecticides in experimental animals have been well documented [11–22]. Regarding the proposed mechanism behind the antidotal action of diphenhydramine, this has been attributed to its potent antimuscarinic [9–11] and possibly antinicotinic effects [23]. However, despite the variations in the effects of diphenhydramine on PChE, EChE, or BChE activities in different animal species intoxicated with OP or CA insecticides [14, 15, 20–22, 31], some studies have suggested that diphenhydramine might protect the enzyme from further inhibition by the insecticides [20–22]. The suggested weak inhibitory action of diphenhydramine on ChE activity [32] might contribute to its antidotal property [21, 22, 30].

This meta-analysis was conducted to resolve the potential beneficial effects of diphenhydramine regarding ChE inhibition. It demonstrated the beneficial effects of diphenhydramine in cases of poisoning by OP or CA insecticides in experimental animals at the level of ChE. This was shown by the combined effect size (high ChE inhibition) being significantly more favorable in the control-untreated (non-diphenhydramine) group; that is, lower ChE inhibition in the diphenhydramine intervention group (Figure-2). However, it should be stressed here that the activities of all three ChEs were significantly inhibited by OP or CA insecticides used in the experimental animals (Table-1). This was noticed in the studies regardless of concurrent diphenhydramine therapy, which was administered either before or after the intoxication by insecticides. In this context, it is impossible to draw definitive conclusions on the final clinical outcome of the extent of ChE inhibition after the diphenhydramine intervention, as the time points of blood or tissue sampling were within a few hours of intoxication. However, the clinical use of diphenhydramine in cases of OP or CA poisoning has been advocated in various animal species [9, 25–29] as well as in humans [23, 24].

This meta-analysis suggested significant heterogeneity (albeit with a moderate I^2^ index of 74%) in the results. Interestingly, subgroup analysis demonstrated no heterogeneity with BChE (I^2^ = 5%), while PChE and EChE had high heterogeneity (I^2^ = 84% and 78%, respectively). It is plausible that the heterogeneity was attributable to the inherent variations in ChE activities found in the plasma, erythrocytes, and brain [42–44]. BChE activity is most closely related to the actual clinical situation of OP or CA poisoning, which results in enzyme inhibition and subsequently produces the characteristic toxidrome of antiChE poisoning [4–6, 42]. Furthermore, supporting the importance of BChE, subgroup analysis revealed that the weight of BChE inhibition in the analysis was 81%, compared with 12% and 7% for PChE and EChE, respectively (Figure-4). However, studies of BChE used in this meta-analysis were conducted on whole-brain homogenate; there is thus a need for detailed studies about the combined effects of diphenhydramine and OP or CA insecticides in different parts of the brain, which are involved in the insecticides’ toxic outcomes [4–6]. Furthermore, all of the studies measured enzyme activities in the blood or the brain by a modified electrometric method [44], thus eliminating the factor of methodological variation that could influence the outcome measures [43]. However, other sources of heterogeneity could be the types of insecticides used, since they variably and differentially inhibit ChEs, and the species of experimental animals, since they inherently possess ChEs of various physiological activities in the blood or brain [43, 44]. In support of our meta-analysis, which was conducted on blood and brain ChEs *in vivo* (mice, rats, and chicks), *in vitro* studies on human PChE and EChE [30] and chick PChE and BChE [21, 22] suggested that diphenhydramine might partially protect the enzyme from additional inhibition induced by OP or CA insecticides. However, at the current stage, we cautiously draw such a conclusion, since the prediction of a future study by the drapery plot suggested the possibility of a shift of the point of effect size toward the diphenhydramine intervention. There is thus a need for additional studies on the effects of diphenhydramine following intoxication with various OP and CA insecticides in experimental animals.

Visual examination of the funnel plot together with results of the objective Egger’s test indicated an absence of publication bias in the outcome covering the studies under this meta-analysis. In the contour-enhanced funnel plot, there was a symmetrical distribution of the effect size points (Figure-5). This obviated the need for trim-and-fill analysis in the funnel plot [40, 41].

## Conclusion

This meta-analysis suggests that, in addition to its documented antidotal properties against poisoning with OP or CA insecticides, diphenhydramine could also reduce the extent of their ChE inhibition, especially in the brain, which is the main target of toxicity upon intoxication with these insecticides. There is a need for additional studies about the interaction of diphenhydramine with ChE-inhibiting insecticides in different parts of the brain, which are crucial in cases of poisoning with these insecticides.

## Authors’ Contributions

FKM: Conceptualized the study and drafted the manuscript. FKM, HMSG, AAM and HMR shared equally in the review and meta-analysis, provided comments, participated in writing the manuscript, and approved the final version of the manuscript.
